# Endophytic *Beauveria bassiana* in Foliar-Treated *Citrus limon* Plants Acting as a Growth Suppressor to Three Successive Generations of *Diaphorina citri* Kuwayama (Hemiptera: Liviidae)

**DOI:** 10.3390/insects10060176

**Published:** 2019-06-19

**Authors:** Bamisope Steve Bamisile, Chandra Kanta Dash, Komivi Senyo Akutse, Muhammad Qasim, Luis Carlos Ramos Aguila, Fangfei Wang, Ravindran Keppanan, Liande Wang

**Affiliations:** 1 State Key Laboratory of Ecological Pest Control for Fujian and Taiwan Crops, College of Plant Protection, Fujian Agriculture and Forestry University, Fuzhou 350002, China; luisramosaguila@gmail.com (L.C.R.A.); stephaniefangfei@yahoo.com (F.W.); 2 Plant Protection College, Fujian Agriculture and Forestry University, Fuzhou 350002, China; 3 Institute of Applied Ecology and Research Centre for Biodiversity and Eco-Safety, Fujian Agriculture and Forestry University, Fuzhou 350002, China; 4 Department of Entomology, Faculty of Agriculture, Sylhet Agricultural University, Sylhet 3300, Bangladesh; chandra.ento.sau@hotmail.com; 5 International Centre of Insect Physiology and Ecology, P.O. Box 30772-00100 Nairobi, Kenya; kakutse@icipe.org; 6 Ministry of Agriculture Key Lab of Molecular Biology of Crop Pathogens and Insects, Institute of Insect Science, Zhejiang University, Hangzhou 310058, China; qasi.sas@gmail.com; 7 Department of Entomology and Plant Pathology, Faculty of Agriculture, Chiang Mai University, Chiang Mai 50200, Thailand; ravikindran07@yahoo.com

**Keywords:** fungal endophytes, endophytic colonization, plant growth, survival, Asian citrus psyllid, biological control

## Abstract

Entomopathogenic fungi are commonly applied as inundative sprays to protect plants against insect pests. Their artificial establishment as fungal endophytes to provide other benefits to the host plants aside mere protection against the primary pests has also been widely demonstrated. In the present study, two fungal strains of *Beauveria* *bassiana* and one strain of *Isaria fumosorosea* were assessed in a pathogenicity test against adults of Asian citrus psyllid (*Diaphorina citri*) and found to induce 50% reduction in the survival rate of *D. citri* adults within 5 days of exposure. The ability of the three fungal strains to endophytically colonize *Citrus limon*, the impact on plant growth and the effects of systemic colonization on 3 successive generations of *D. citri* feeding on colonized plants was evaluated. Citrus seedlings at 4 months post-planting were inoculated with each of the fungal strains via foliar spraying. Both strains of *B. bassiana* successfully colonized the seedlings. One of the *B.* *bassiana* strains (BB Fafu-13) was sustained up to 12 weeks in the colonized seedlings, whereas the other *B. bassiana* strain (BB Fafu-16) was only recovered up to 8 weeks post-inoculation. *Isaria* *fumosorosea* (IF Fafu-1) failed to colonize the plant. Both strains of *B. bassiana* induced significant improvement in plant height and flush production in endophytically colonized seedlings. In addition, endophytic *B. bassiana* caused 10–15% *D. citri* adult mortality within 7 days of exposure. Female *D. citri* feeding on *B. bassiana* challenged plants laid fewer eggs as compared to those feeding on endophyte-free seedlings, while reduction in adult emergence was recorded on *B. bassiana* treated plants. With this study, we present the first evidence of *B.* *bassiana* artificial establishment as fungal endophyte in citrus plants and its negative effects on *D. citri*.

## 1. Introduction

*Citrus limon* and the rest of other citrus varieties are fruit crops of huge economic importance around the world in lieu of their popularity, availability, nutritional value, contribution to foreign exchange and industrialization [1]. However, the damage done by the Asian citrus psyllid *Diaphorina citri* Kuwayama (Hemiptera: Liviidae) and its transmitted disease pathogen—*Candidatus* Liberibacter asiaticus (C*las*)—is becoming a serious threat to citrus production across the globe [2]. The most commonly used control method for *D. citri* has been chemical insecticides [3]. However, persistent use of these insecticides has resulted in the development of resistance by the pests, as well as posing environmental and economic hazards [4]. This problem has brought about the need to introduce other sustainable, effective, and safe alternative control strategies against *D. citri* [5].

Over the years, various biological control tactics have been evaluated in many parts of the world for the management of this pest. The most common classical biological control method is the use of parasitoids. The specialist wasp: *Tamarixia radiata* (Waterston) (Hymenoptera: Eulophidae) and *Diaphorencyrtus aligarhensis* (Shaffee, Alam and Agarwal) (Hymenoptera: Encyrtidae) are the notable examples [6]. Entomopathogenic fungi in form of inundative sprays have been commonly used in various trials against *D. citri* thus far [7,8,9]. However, the exposure of fungal spores to environmental conditions such as UV, temperature, and humidity reduce the efficacy of entomopathogenic fungi [5]. There is a general opinion that entomopathogenic fungi as fungal endophytes could cope better with these negative environmental factors [10].

The inoculation of entomopathogenic fungi into crop plants to establish as fungal endophytes has brought about a unique dimension into insect pests’ management programmes [11,12]. A typical example is *Beauveria bassiana* (Balsamo) Vuillemin (Hypocreales: Cordycipitaceae), which has a wide-range of host plant species and a worldwide distribution, making it the most abundant species of the numerous entomopathogenic fungal species reported till date [13]. *Beauveria bassiana* has been reported as naturally occurring in many plant species, for instance, in corn and cotton [14], coffee [10], cocoa [15], Ironwood [16], western white pine [17], and jimsonweed [14]. In addition, re-isolation of *B. bassiana* from different plant species following its artificial inoculation has been reported in corn [18], tobacco [19], wheat [20], durum wheat [21], coffee [22,23], faba bean [24], common bean [25], grapevine [26], and soybean [27]. *Isaria fumosorosea* Wize (Hypocreales: Cordycipitaceae) on the other hand, is not as common in nature as *B. bassiana*. However, the fungus has also been reported as plant tissues colonizer, plant growth enhancer, or as a naturally-occurring endophyte in some previous studies [25,28,29].

Plant colonization by various species of entomopathogenic fungi has been reported to improve the growth of the plants, reduce the damage caused by many plant herbivores, and cause a reduction in the growth rate and reproduction of a wide range of insect pests of various feeding guilds including weevils, beetles, aphids, mites, and caterpillars. This approach has been tested successfully and found effective in numerous economic crops [20,24,25,30,31,32,33,34]. There is evidence that the practice of using naturally occurring microorganisms over time could serve as a suitable replacement for chemical control method, which is the most commonly used technique for pest control across different ecological zones [35,36,37].

However, to the best of our knowledge, there are no reports on the establishment of entomopathogenic fungi as endophytes in *Citrus* spp. for the control of citrus insect pests. The potential of entomopathogenic fungi successfully colonizing *C. limon* seedlings, sustenance of endophytic colonization in plant over time, improvement of plant growth and inducing negative effects on the rate of survival, growth and reproduction of *D. citri* would help offer an alternative approach to the common practice of direct spraying of insect pests or citrus trees with entomopathogenic fungi. Hence, the present study investigated the ability of *B. bassiana* and *I. fumosorosea* to endophytically colonize *C. limon* and the impact on seedling growth. We also evaluated the effects of systemic endophytic colonization by entomopathogenic fungi on three successive generations of *D. citri* feeding on endophytically colonized citrus seedlings.

## 2. Materials and methods

### 2.1. Source of Fungi

*Beauveria bassiana* strains (BB Fafu-13 and BB Fafu-16) and *I. fumosorosea* (IF Fafu-1) used in this study were obtained from the fungal culture bank of Fujian Agriculture and Forestry University, Fuzhou, Fujian, China PR. The fungi were originally isolated from mycosed *D. citri* adults collected from a private citrus orchard in *Rixi*, a satellite town of Fuzhou, Fujian province, South Eastern part of China (26°21´8.88´´N, 119°16´9.52´´E). The fungal strains were sub-cultured on potato dextrose agar (PDA, Qingdao Hope Bio-Technology Co., Ltd. Qingdao, China), and stored at 25 °C in an incubator. All isolates (BB Fafu-13, BB Fafu-16 and IF Fafu-1) were identified morphologically using the taxonomy keys of Humber [38,39], and by using sequence data from the ITS region of nuclear rRNA (GenBank accession numbers MG844430 for BB Fafu-13, MG844431 for BB Fafu-16, and MG837716 for IF Fafu-1). The strains were selected for the current study based on the source and the findings of our previous studies [25,40].

### 2.2. Source of Psyllids for Laboratory Assays

Asian citrus psyllid adults were collected from naturally occurring populations growing on *Citrus limon* trees in a private orchard in *Rixi*, Fuzhou, China between July and September 2016. *D. citri* adults were reared in greenhouse on *C. limon* seedlings at 25 (±2 °C) and 65–75% relative humidity (RH), under a 14 h light:10 h dark (L:D) photoperiod, for up to 5 successive generations prior to the experiment. Adult psyllids used for the bioassay were 0–3 days old, obtained by transferring adults from the stock population to fresh *C. limon* seedlings. Females were allowed to oviposit for 3 days and then removed to allow collection of newly emerged adults for the bioassay experiments.

### 2.3. Citrus Plants

Citrus seedlings (*C. limon* var. Sichuan *an yue*) used in the experiment were raised from surface-sterilized seeds washed in 70% ethanol for 3 min and 2% sodium hypochlorite for 3 min and then rinsed thrice in sterile distilled water. Surface sterilized seeds were planted in pollen trays (40 × 30 × 5 cm) containing sterilized compost, vermiculture and garden soil mixes. Soil mixes were sterilized twice in an autoclave for 2 h at 120 °C at each sterilization time, giving a 24 h interval between each sterilization and allowing soil to cool for 72 h before use. Seedlings were transplanted at 40 days post-planting into 12 × 12 × 10 cm seedling pots filled with sterilized compost, garden soil and vermiculture at 5:4:1 ratio. Transplants were grown in a glasshouse at 25 (±2 °C) and 65–75% relative humidity (RH), under a 16 h light:8 h dark (L:D) photoperiod. Seedlings were watered at 5–6 days interval with sterile distilled water and fertilized monthly using 1 g L^−1^ Basfoliar 30-10-10+Mg+TE (COMPO Expert GmbH, Krefeld, Germany) inorganic fertilizer.

### 2.4. Preparation of Conidial Suspensions

Three fungal strains of *B. bassiana* and *I. fumosorosea* were evaluated in a preliminary in vitro pathogenicity test against *D. citri*. The conidia used for the bioassay were harvested from 14-day old cultures grown on potato dextrose agar (PDA) at 25 °C in an incubator. The conidia were harvested by scrapping the fungal mycelia from the surface of the agar using a sterile scalpel under axenic conditions. The harvested conidia were then suspended in 20 mL 0.01% Tween 80 solution, vortexed for 3 min and then filtered using a sterile syringe and cotton wool to remove hyphal debris to obtain a clean stock suspension. The conidia concentration of the stock fungal suspension was determined by preparing a 10,000-fold serial dilution to enable conidial count, where 100 μL of the stock suspension was diluted with 900 μL of sterile distilled water containing 0.01% Tween 80 solution, and then vortexed for 30 s and the procedure was repeated for the next dilution. The inoculum was prepared by adjusting the initial stock concentration to a final concentration of 1 × 10^8^ conidia mL^−1^ using the Neubauer hemocytometer [41]. Following the procedure of Russo et al. [42], a conidial germination test was conducted to determine the conidial viability of all fungal strains. 

### 2.5. Pathogenicity Test against D. citri Adults

Fungal bioassay experiment was conducted in the greenhouse, where 20 newly-emerged adults of *D. citri* (0–3 days old) were maintained on each *C. limon* seedling. Each seedling with 20 adults of *D. citri* were sprayed with 10 ml of 1 × 10^8^ conidia mL^–1^ of each conidial suspensions (three seedlings per treatment). The control seedlings with 20 *D. citri* adults each (in three replications) were sprayed with sterile distilled water containing 0.01% Tween 80 solution. A total of 240 adults of *D. citri* were treated in the whole experiment. The rate of mortality was assessed daily for 9 days post-exposure. Daily, individual adults were touched using a paintbrush one after the other, insects that did not respond to touch were recorded as dead insects. To confirm that psyllids mortality resulted from fungal infection, dead insects were placed on a moistened filter paper in Petri dishes and observed for post-mortem fungal sporulation.

### 2.6. Inoculation of Citrus Plants

The experiment was conducted following a complete randomized design with three fungal strains and a control; each treatment with 20 seedlings. At 14 weeks post-transplanting date (see Section 2.3), the leaves of the plants were sprayed with an average of 10 mL of 1 × 10^8^ conidial mL^–1^ suspension of each fungal strain or sterile distilled water containing 0.01% Tween 80 solution as control. The inoculated leaves were counted and marked, while the plant height was measured. Seedlings were covered with nylon bags for 24 h to conserve humidity [41]. Seedlings were kept in a glasshouse at 25 (± 2 °C) and 65–75% relative humidity (RH), under a 14 h light:10 h dark (L:D) photoperiod until assessment for endophytic colonization was done. 

### 2.7. Effects of Endophytic Entomopathogenic Fungi Inoculation on Plant Growth

For examining the effects of foliar inoculation with entomopathogenic fungi on citrus seedlings growth, five seedlings were randomly selected per treatment, whereby the height of the seedlings (the distance from plant base to the tip of the stem) were measured, and the number of leaves were counted at 4, 8, and 12 weeks post-inoculation (wpi).

### 2.8. Assessment of Endophytic Colonization

At 4, 8, and 12 weeks post foliar inoculation of citrus plants with fungal entomopathogens, endophytic colonization by the inoculated fungal strains was determined. To assess plant for systemic colonization, the newly emerged plant parts (stems and leaves) that developed after foliar spraying with entomopathogenic fungi were separated from the marked plant parts that were directly sprayed with fungal conidia (see Section 2.6). The assessment was done by carefully uprooting four seedlings (per treatment) from the soil, washing the plants under tap water, and then, cutting the plant tissues into five sections, that is: upper leaves, lower leaves, upper stems, lower stems, and roots. The upper leaves and upper stems are young/unmarked plant parts that emerged post-inoculation, while lower leaves and lower stems are old/marked plant parts that were directly sprayed with fungal conidia suspension at inoculation date. Plant tissues cutting was followed by surface sterilization in 70% ethanol for 3 min and 2% sodium hypochlorite for 3 min, and then rinsed thrice in sterile distilled water [43]. The plant tissues were air-dried on a sterile tissue paper. The outer edges of the plant parts were trimmed off, as the endophytes in this region might have been eliminated due to exposure to disinfectants during surface sterilization. The plant tissues were further trimmed into smaller sections of about 8 × 8 mm long and plated on freshly prepared PDA in 9.0 cm Petri dish. Streptomycin sulfate and chloramphenicol at 1.25 g L^−1^ were added to suppress bacterial contamination. Six tissue pieces were collected per section and plated on each Petri dish and sealed with parafilm. To determine the success of the surface sterilization procedure, 100 μL of the last rinsed water was plated on agar and incubated at 25 °C for 7 days [44]. Tissue imprint method was also used to confirm the success of sterilization techniques, where leaf discs were imprinted on the surface of standard PDA plates [45]. The corresponding samples will not be considered for analysis if fungal growth is recorded on the plate. The Petri dishes containing the plant tissues were stored at 25 °C in an incubator and inspected periodically at 2–3 days interval to assess them for fungal growth. Plant parts that exhibit fungal growth were recorded and transferred into fresh PDA plates as to prevent contamination. Emerging fungal growth recorded from each plant parts were morphologically identified by comparing the mycelia and growth pattern with the mother culture and by classical microscopic observation and taxonomy keys in line with Humber [38,39]. This was done by viewing the conidia and conidiophores with the help of a light microscope (Model CX21FS1—Olympus Corporation, Tokyo, Japan).

### 2.9. Effects of Endophytically-Colonized Citrus Plants on the Development and Fecundity of Three Successive Generations of the Asian Citrus Psyllid

To assess the systemic effects of tested *B. bassiana* strains on the fecundity and development of *D. citri*, citrus seedlings were foliar-sprayed with an average of 10 mL of 1 × 10^8^ conidial mL^–1^ suspension of each *B. bassiana* strains (BB Fafu-13 and BB Fafu-16), while the control seedlings were sprayed with sterile distilled water containing 0.01% Tween 80 solution.

At 14 days post-inoculation, a single leaf per each seedling was randomly selected and excised to assess plants for endophytic colonization by the treated entomopathogenic fungi. Assessment was done using the same method as described in 2.8 above. Five seedlings each were selected per treatment, following confirmation of endophytic colonization by *B. bassiana.* Prior to *D. citri* release, the seedlings were carefully and thoroughly washed to remove any active *Beauveria* spores from the phylloplane and the success of the hand washing procedure was subsequently assessed following the method described by Barta [46]. Adults used in this experiment were raised following the same procedure as described in Section 2.2 above. Twenty newly emerged *D. citri* adults (8 male and 12 female) were introduced per seedlings. Adults were allowed to feed and oviposit for 7 days, during the period, adult mortality was observed and recorded. At 7 days post *D. citri* release, all surviving adults and nymphs (where applicable) were carefully removed using a paintbrush and discarded. Total eggs laid were carefully counted using a 20 × magnification hand lens—Inpelanyu Model C1390-01 (Product of China PR.). The eggs were monitored over a period of 21 days, while taking periodic records of emergence of the first-generation nymphs and adults. Assessment was done at 3–4 days interval. At 21 days post-exposure, all surviving adults and nymphs were again carefully removed and the total eggs laid were counted as before. The eggs were again observed for 21 days as before, for the emergence of second-generation nymphs and adults. The same procedure was repeated to record the third-generation nymphs and adult psyllids. All the experiments were independently conducted twice.

### 2.10. Statistical Analysis

All data collected were subjected to normality and homogeneity test of variances using qqplot, Levene’s homogeneity test and Shapiro–Wilk Normality test prior to any data analysis. In other to stabilize the variance, the colonization percentage, cumulative mortality percentage, nymph, and adult emergence values were subjected to log transformation before any statistical analysis was done. In the in vitro bioassay experiment for *D. citri*, the rate of survival was computed using the Kaplan–Meier method and compared using the log-rank test. Data of adult cumulative mortality, fecundity, nymph and adult emergence were analyzed using a repeated-measures one-way ANOVA with the assessment time as a repeated factor, while plant growth data were subjected to one-way ANOVA. Comparisons between treatment means were performed using the least significance difference (LSD) test. Data were expressed as means ± standard error (SE) and statistical significance was set at <0.05. 

The colonization frequency of each endophytic entomopathogenic fungus was determined using the formula of Petrini and Fisher [47] as modified by Posada et al. [23] and Klieber and Reineke [31] as: colonization frequency = 100 × (number of plants from which fungal endophyte was re-isolated/total number of plants treated with entomopathogenic fungi). All the statistical analyses were performed using IBM SPSS statistical software v22.0 (SPSS Inc., Chicago, IL, USA) and Statistix 8.1 (Analytical Software, Tallahassee, FL, USA).

## 3. Results

### 3.1. Pathogenicity of Entomopathogenic Fungal Strains against D. citri Adults

**Figure 1:**
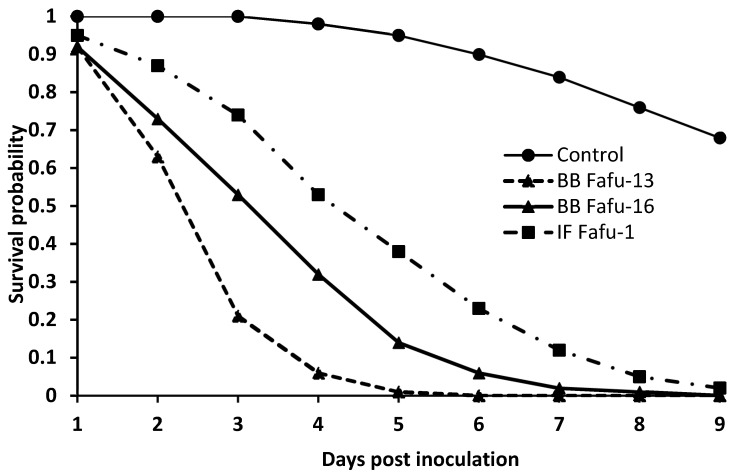
Graph showing survival curves of Asian citrus psyllid adults exposed to *Beauveria bassiana* BB Fafu-13, BB Fafu-16, *Isaria fumosorosea* IF Fafu-1 and the control at different days post-exposure (1–9 days).

The viability test resulted in over 95% germination of the conidia of all fungal strains. Sixty (60) *D. citri* adults were exposed to each of the three entomopathogenic fungal strains or distilled water containing 0.01% Tween 80 as control. All fungal strains induced decrease in survival rate of adult psyllids. At 2 days post-exposure, the mean survival was 68%, 80%, 92%, and 100% for BB Fafu-13, BB Fafu-16, IF Fafu-1, and control, respectively. Within 5 days of exposure to fungal entomopathogens, mean survival declined to less than 50% in all fungal treatments, while mean survival was 95% in the control. BB Fafu-13 was the most virulent of the treated entomopathogenic fungal strains, as 2% mean survival was recorded at 8 days post-exposure, which was significantly lower than other treatments. Using the Kaplan–Meir method, the survival probability of *D. citri* adults varied among the treatments (proximate log-rank test = 101.08, df = 3, *p* < 0.001) (Figure 1).

### 3.2. Assessment of Systemic Endophytic Colonization of Citrus Seedlings by Entomopathogenic Fungi 

**Figure 2:**
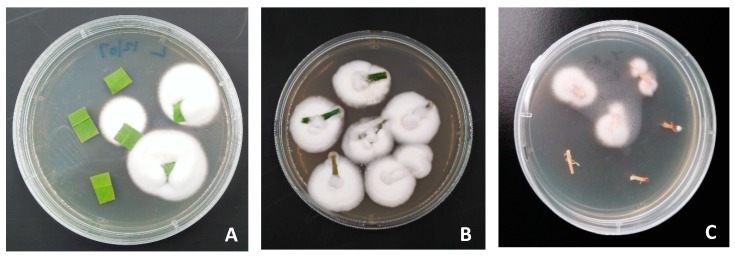
Endophytic entomopathogenic fungi (*Bassiana bassiana* BB Fafu-13) re-isolation from (**A**) leaf, (**B**) stem, and (**C**) root tissues of *Citrus limon* plant following foliar treatment with fungal conidia suspension.

Two of the three entomopathogenic fungal strains examined in this study, *B. bassiana* (BB Fafu-13) and *B. bassiana* (BB Fafu-16), successfully colonized various citrus plant parts (leaves, stems, and roots) following foliar spraying with conidial suspensions of each fungal strain. The re-isolation of individual fungal species from the tissues of treated plants that were plated on PDA confirmed the endophytic colonization of the different plant parts by *B. bassiana* strains, BB Fafu-13 (Figure 2) and BB Fafu-16. However, *I. fumosorosea* IF Fafu-1 failed to colonize the citrus plant. Surface sterilization technique for the plant tissues prior to plating on PDA was effective, as the last rinse distilled water and tissue imprints did not yield any fungal growth on PDA. BB Fafu-13 was recovered from all plant parts (leaves, stems, and roots), however, re-isolation from the root tissues was only possible at 12 wpi. From BB Fafu-16 treated seedlings, fungal endophyte was only re-isolated from the leaf and stem tissues, but never from the root. No fungi re-isolation was recorded in IF Fafu-1 treated and the untreated control seedlings. Endophytic colonization was sustained in BB Fafu-13 treated seedlings up to 12 wpi, whereas, no endophytic fungi recovery was recorded in BB Fafu-16 treated seedlings after 8 wpi.

Possible migration of the endophytes within the endophytically colonized seedlings from treated plant parts to other emerging plant tissues post-inoculation was assessed. Both *B. bassiana* strains that successfully colonized *C. limon* plants were observed to migrate from treated plant parts to newly emerged plant tissues, as endophytic fungi were re-isolated from the upper leaves and stems of BB Fafu-13 and BB Fafu-16 treated seedlings. At 4 and 8 wpi, 50% and 25% of the upper leaves, respectively, were colonized by both strains, while 25% of the upper stems were colonized by both strains at 4 wpi. However, at 12 wpi, re-isolation was only recorded in BB Fafu-13 treated seedlings (Table 1).

### 3.3. Effects of Endophytic Fungal Strains on Seedling Growth

Endophytically colonized citrus seedlings were assessed for the potential of the fungal endophytes improving seedling height and number of leaves. The seedlings growth assessment was done at 4, 8, and 12 wpi. Generally, increase in height recorded across all treated seedlings at week 4 (F_3,16_ = 1.96, *p* = 0.1609) and 8 (F_3,16_ = 2.30, *p* = 0.1167) was relatively similar to the untreated control seedlings. However, data collected at 12 wpi showed that height gained in BB Fafu-16 and BB Fafu-13 treated seedlings were significantly higher than untreated control seedlings (F_3,16_ = 4.36, *p* = 0.0200; Table 2). On the other hand, both strains of *B. bassiana* (BB Fafu-13 and BB Fafu-16) that resulted in successful endophytic colonization of the inoculated citrus seedlings appeared to induce flush production, as significant differences were recorded among the treatments with regards to the number of leaves counted at individual sampling dates (4; F_3,16_ = 3.33, *p* = 0.0463, 8; F_3,16_ = 3.09, *p* = 0.0503 and 12 wpi; F_3,16_ = 3.55, *p* = 0.0384; Table 2).

### 3.4. Assessment of the Effects of Endophytically-Colonized Citrus Plants on the Development and Fecundity of Three Successive Generations of the Asian Citrus Psyllid

**Figure 3:**
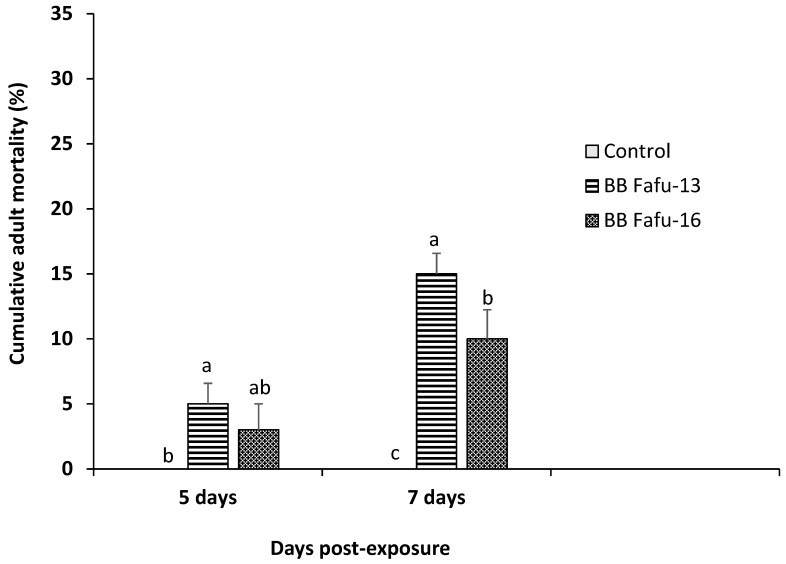
Cumulative adult mortality percentage of *Diaphorina citri* at 5- and 7-days post-exposure to treatment with *Citrus limon* plants challenged by endophytic *Beauveria bassiana* (BB Fafu-13 and BB Fafu-16) and the control. Bars (±SE) with different letters within the same assessment date indicate significant differences among treatments at *p* = 0.05 (LSD after repeated-measures One-way ANOVA).

**Figure 4:**
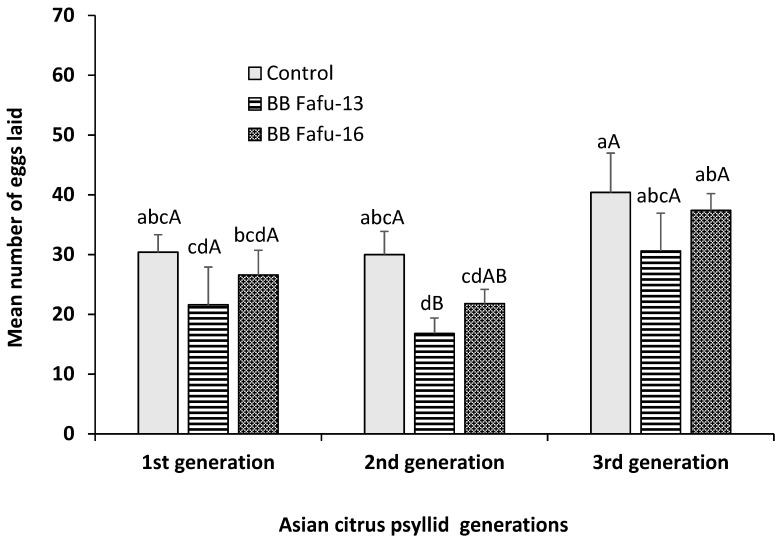
Mean number of eggs laid (± SE) by *Diaphorina citri* in 3 successive generations in response to treatment with *Citrus limon* plants challenged by endophytic *Beauveria bassiana* (BB Fafu-13 and BB Fafu-16) and the control. For each generation, bars (±SE) with different uppercase letters indicate significant differences among treatments. Different lowercase letters show significant differences across the generations at *p* = 0.05 (LSD after repeated-measures One-way ANOVA).

The development and fecundity of Asian citrus psyllid adults feeding on endophyte-challenged and endophyte-free seedlings was observed over three successive generations. Endophytic *B. bassiana* induced significant increase in the rate of mortality of *D. citri* adults feeding on colonized *C. limon* seedlings. No *D. citri* adult mortality was recorded across all treatments and control until 5-days post-exposure, therefore, only data collected at 5- and 7-days post-exposure were indicated (Figure 3). Cumulative mortality percentage recorded at 5- and 7-days post-exposure to endophytic colonized seedlings was 5.0 ± 1.6, 3.0 ± 2.0, 0.0 ± 0.0, and 15.0 ± 1.6, 10.0 ± 2.2, 0.0 ± 0.0 for BB Fafu-13, BB Fafu-16, and control respectively. Both strains of *B. bassiana* significantly increase mortality rate of *D. citri* adults at 5 (F_2,8_ = 5.46, *p* = 0.0319) and 7 days post exposure (F_2,8_ = 20.0, *p* < 0.001; Figure 3). With respect to fecundity, mean number of eggs laid by the first generation female *D. citri* was relatively similar among treatments and control (F_2,8_ = 1.08, *p* = 0.3855), however, there was a significant reduction in the mean number of eggs laid by the second generation female *D. citri* feeding on endophyte-challenged seedlings as against the control (F_2,8_ = 4.85, *p* = 0.0286). In contrast, the third generation female *D. citri* appeared to be less affected by the endophytic *B. bassiana,* as similar mean number of eggs were recorded across both *B. bassiana* colonized seedlings and the control (F_2,8_ = 1.50, *p* = 0.2795; Figure 4). 

Endophytic *B. bassiana* mediated reduction in nymph and adult psyllids emergence across the three successive generations. Both strains of *B. bassiana* induced a significant reduction in number of nymphs at each assessment dates (7, 10, 14, 17, and 21 days post-exposure) across all three generations (Table 3). In addition, significantly lower mean number of adults were recorded on the seedlings colonized by the two strains of *B. bassiana* in comparison with the control seedlings for the first (F_2,8_ = 5.07, *p* = 0.0379), second (F_2,8_ = 5.36, *p* = 0.0334), and third generations (F_2,8_ = 4.50, *p* = 0.0491, Table 3). 

## 4. Discussion

We demonstrated that entomopathogenic fungi *B. bassiana* and *I. fumosorosea* are potential biocontrol agents for the management of Asian citrus psyllid, *D. citri*, expanding the range of studies reporting fungal entomopathogens as an alternative measure for *D. citri* management. We found an increase in mortality rate of *D. citri* treated in vitro with entomopathogenic fungi, which was significantly higher across the treatments compared to the control. All examined entomopathogenic fungal strains induced over 50% *D. citri* adult mortality within 5 days of exposure. Our findings are in line with the studies of Gandarilla-Pacheco et al. [48] and Zhang et al. [49], who reported *B. bassiana* to be pathogenic against *D. citri*. In addition, *I. fumosorosea* has also been evaluated in various studies and the findings revealed a reduction in the survival and developmental rate of *D. citri* post-exposure to the fungus [7,8,9].

The ability of *B. bassiana* to colonize *C. limon* plants endophytically following artificial inoculation was demonstrated in the present study. Two strains of *B. bassiana* evaluated in this study were successfully established as endophytes in *C. limon* plants. The removal of epiphytes from the surface of the plated plant tissues through surface sterilization, non-emergence of *B. bassiana* from any of the control plants and the success of the surface sterilization method following plating of the last rinsed cleaning water and tissue imprints, are evidences to suggest that the trial was successful.

The results showed systemic colonization of the various citrus plant parts: leaves, stems, and roots by *B. bassiana* BB Fafu-13 strain. This is evident in the re-isolation of fungal endophytes from plant parts other than the treated parts. The two fungal strains that successfully colonized the plant might have been translocated across the plant to other tissues (new stems and roots) together with photosynthates as previously reported by Bing and Lewis [18]. Our results further are in line with previous studies suggesting that inoculated fungi have the potential to move across the interconnected vascular tissues, thereby systemically colonizing the entire plant [50,51]. However, our findings contrast with the reports of Jia et al. [52] and Posada et al. [23], who reported temporal non-systemic endophytic colonization of treated plants following foliar treatment with *B. bassiana* conidia suspension. Posada et al. [23] suggested that foliar application method is not the ideal artificial inoculation method for colonizing coffee plants. Jia et al. [52], on the other hand, suggested that the antagonism between *B. bassiana* and the contents of rice plant parenchyma tissues may be the reason for temporal non-systemic endophytic colonization of the plant. Resquín-Romero et al. [53] also suggested that, since there exists a very little chance of colonizing the entire plant through foliar treatment with conidia, transient non-systemic colonization is the most likely outcome. The competition with other existing fungi which forms a barrier for the systemic movement of the inoculated fungi from the point of inoculation to distant parts may be the reason for transient non-systemic colonization [54].

Our study showed that *B. bassiana* was retained up to three months in colonized citrus plants. This result is similar to the study of Brownbridge et al. [55] and Posada et al. [23], where *B. bassiana* was reported to be retained in radiata pine and coffee up to nine and eight months respectively. Biswas et al. [56] also reported sustenance of *B. bassiana* up to three months in jute plants. A report of *B. bassiana* and *M. brunneum* colonizing *V. faba* for one month is also available [34].

*Beauveria bassiana* enhanced the growth of inoculated citrus seedlings, as there were significant differences between *B. bassiana* treated seedlings and the untreated control seedlings with respect to the average height of seedlings and number of individual leaves at different weeks post-inoculation. This outcome is in line with several previous studies that presented evidences of fungal endophytes significantly improving the growth of colonized plants [12,30,34,57,58]. For instance, the height, number of leaf pair, fresh weight of shoots and roots of *V. faba* plants inoculated with *B. bassiana, Beauveria brongniartii,* and *M. brunneum* via foliar inoculation were reported to be improved according to Jaber and Enkerli [58]. Similarly, an improvement in growth and dry biomass of cotton plants inoculated with *B. bassiana* and *Purpureocillium lilacinum* has also been reported [57]. In addition, *I. fumosorosea* has also been reported as a plant growth enhancer [25,28,29]. Although the *I. fumosorosea* strain evaluated in this study failed to colonize *C. limon* seedlings, the fungus was successfully established as an endophyte in common bean [25]. The dissimilarity in fungal strains and plant species inoculated might be the factors responsible for this outcome [19,23,24].

Endophytic *B. bassiana* mediated increase in adult mortality, reduction in fecundity rate, as well as, reduction in the rate of nymph and adult emergence. Endophytic *B. bassiana* induced 10% to 15% adult mortality within 7 days of exposure. No mycosis was detected on any of the dead psyllids. The outcome is consistent with previous studies where no fungal outgrowth was discovered in cadavers of insects fed with endophytically colonized plants [21,53]. Several published papers have ascribed the potential of entomopathogenic fungi to cause mortality in insect pests to their ability to synthesize mycotoxins such as beauvericin, bassianolide, oosporein, destruxin, and tenellin [53,59,60]. We found a significant reduction in nymph and adult emergence across three successive psyllid generations. For instance, BB Fafu-13 appeared to induce a decline in the rate of development of *D. citri*, as no adult emergence was recorded at 17 days post *D. citri* exposure to BB Fafu-13 challenged seedlings at the first and second generations. It was observed that all of the nymphs that emerged from the eggs laid by female *D. citri* feeding on BB Fafu-13 challenged seedlings failed to emerge as adults after 17 days, as against the control seedlings, where significantly higher mean number of adult emergences was recorded. Naturally, according to Grafton-Cardwell et al. [61], eggs laid by *D. citri* takes between 2 to 4 days to hatch, while the five instars stages are completed between 11 to 15 days. However, for third generation psyllids, adult emergence was recorded at 17 days post-exposure on BB Fafu-13 challenged seedlings. Generally, we recorded an increase in mean number of nymphs and adults at the third generation across all treatments. The decline in percentage endophytic colonization of seedlings by *B. bassiana* over time that was recorded in this study might be responsible for this outcome. Similarly, Jaber and Araj [12] reported negative effects of endophytic *B. bassiana* on the development of two successive generations of green peach aphid (*Myzus persicae*). Also, Sánchez-Rodríguez et al. [21] established the potential of endophytic *B. bassiana* to increase the mortality rate of cotton leafworm (*Spodoptera littoralis*) larvae up to 57% in treated cotton leaves. Similar outcome was found in the study of Resquín-Romero et al. [53], where 25% to 46.7% mortality of *S. littoralis* larvae was reported following treatment of alfalfa, melon and tomato plants with *B. bassiana*. Also, Rondot and Reineke [26] found a significant reduction in infestation rate and growth of vine mealybug (*Planococcus ficus*) following treatment of grapevine (*V. vinifera*) with endophytic *B. bassiana*. The aforementioned studies evaluated a similar artificial inoculation method as the present study, aside the study of Jaber and Araj [12], where soil drenching method was used. To date, most studies investigating the negative effects of endophytic colonization of plant on primary pests have been carried out using *B. bassiana* [20,24,25,30,31,32].

## 5. Conclusions and Future Research Directions

The ability of biocontrol agents such as entomopathogenic fungi to adapt and survive in environments foreign to their origin is one of the key characteristics that could make them very effective and generally acceptable for use. The successful establishment of *B. bassiana* as an endophyte in *C. limon* plants and its negative endophytic effects on *D. citri*, as reported for the first time in this study, provides an alternative management strategy to the conventional practice of inundative spraying of biopesticides in tree crops and against insect pests of citrus. Future studies will focus on evaluating the mechanisms underlying the systemic effect of these fungal endophytes on *D. citri* life parameters, as well as, assessing other entomopathogenic fungal species for their potential use in the form of fungal endophyte in citrus pests’ management.

## Figures and Tables

**Table 1:** Colonization frequency of endophytic entomopathogenic fungi in *Citrus limon* plants segments (leaf, stem and root) at different weeks post inoculation (wpi) following foliar treatment of seedlings with 1 × 10^8^ conidia mL^−1^ of each of the treated *Beauveria bassiana* isolates (BB Fafu-13 and BB Fafu-16).

Treatments		Colonization Frequency in Different Plant Segments									
			Leaf				Stem				Root	
			UL		LL		US		LS			
	n **	+	%	+	%	+	%	+	%	+	%
**4 wpi**											
BB Fafu-13	4	2	50	3	75	1	25	2	50	0	0
BB Fafu-16	4	2	50	3	75	1	25	1	25	0	0
**8 wpi**											
BB Fafu-13	4	1	25	3	75	0	0	2	50	0	0
BB Fafu-16	4	1	25	2	50	0	0	1	25	0	0
**12 wpi**											
BB Fafu-13	4	1	25	2	50	0	0	2	50	3	75
BB Fafu-16	4	0	0	0	0	0	0	0	0	0	0
**Total**	24	7	29.2	13	54.2	2	8.3	8	33.3	3	12.5

IF Fafu-1 treated seedlings were not represented as no endophytic fungi re-isolation was recorded. n ** There were four replicates for each fungal isolate at different assessment dates. Four different plants were examined for endophytic colonization, and within each plant, upper leaves (UL), lower leaves (LL), upper stems (UP) lower stems (LS), and roots were separately assessed. Six surface-sterilized leaf discs, stem, and root cuttings were obtained per plant segments (total of 30 sections per plant). Provided fungal endophyte was recovered from one of the six discs or cuttings obtained from a single plant, the plant was assumed to have been endophytically colonized. + Number of plants from which endophytic fungi was re-isolated out of the 4 plants examined (at least 1 of the 6 discs or cuttings showed fungal outgrowth). %—colonization frequency percentage (number of plants from which endophytic fungi was re-isolated ÷ total number of plants assessed × 100). UL—upper leaves and US—upper stems are unmarked plant parts that emerged post inoculation. LL—lower leaves and LS—lower stems are marked plant parts that were directly sprayed with fungal conidia suspension at inoculation date.

**Table 2:** Mean (±SE) plant growth parameters (height and leaf number) of citrus seedlings at different weeks post-inoculation (4, 8, and 12) with *Beauveria bassiana* BB Fafu-13, BB Fafu-16, *Isaria fumosorosea* IF Fafu-1 and untreated control.

Treatments	Seedling Height (cm)			Leaf Number		
	4 wpi	8 wpi	12 wpi	4 wpi	8 wpi	12 wpi
BB Fafu-13	30 ± 1.5a	38 ± 2.5a	44 ± 2.9a	29 ± 1.2a	43 ± 1.4a	54 ± 2.5a
BB Fafu-16	27 ± 0.9a	37 ± 1.4ab	41 ± 1.3ab	25 ± 1.2ab	41 ± 1.2ab	52 ± 1.4a
IF Fafu-1	29 ± 1.0a	34 ± 1.7ab	38 ± 2.2bc	24 ± 2.0b	39 ± 1.7b	48 ± 3.0ab
Control	27 ± 0.4a	32 ± 1.5b	34 ± 1.5c	23 ± 0.7b	37 ± 1.4b	44 ± 1.7b

Means (±SE) with different letters within the same column (same wpi and growth parameter) indicate significant differences among treatments at *p* < 0.05. (LSD after One-way ANOVA).

**Table 3:** The effects of endophytic *Beauveria bassiana* isolates (BB Fafu-13 and BB Fafu-16) on the development of three successive generations of Asian citrus psyllid (*Diaphorina citri*) assessed at different days (7, 10, 14, 17, and 21 days) post exposure to endophyte-challenged *Citrus limon* seedlings.

Days Post Exposure	Treatments	Number of *D. citri* Nymphs and Adults					
			1st Generation Psyllids		2nd Generation Psyllids		3rd Generation Psyllids	
		Nymphs	Adults	Nymphs	Adults	Nymphs	Adults
7 days	Control	5.2 ± 1.4a	0.0 ± 0.0	4.4 ± 0.9a	0.0 ± 0.0	5.2 ± 1.3a	0.0 ± 0.0
	BB Fafu-13	1.0 ± 0.6b	0.0 ± 0.0	1.0 ± 0.4b	0.0 ± 0.0	1.6 ± 0.6b	0.0 ± 0.0
	BB Fafu-16	1.6 ± 0.5b	0.0 ± 0.0	1.2 ± 0.5b	0.0 ± 0.0	1.4 ± 0.4b	0.0 ± 0.0
10 days	Control	13.2 ± 2.5a	0.8 ± 0.5a	12.2 ± 2.9a	0.8 ± 0.5a	12.0 ± 2.0a	0.2 ± 0.2a
	BB Fafu-13	1.2 ± 0.8b	0.0 ± 0.0a	1.2 ± 0.6b	0.0 ± 0.0a	5.4 ± 0.7b	0.0 ± 0.0a
	BB Fafu-16	3.2 ± 0.7b	0.0 ± 0.0a	1.4 ± 0.6b	0.0 ± 0.0a	6.6 ± 0.6b	0.0 ± 0.0a
14 days	Control	24.0 ± 4.0a	9.4 ± 2.0a	16.4 ± 3.0a	11.6 ± 2.1a	19.4 ± 3.1a	12.2 ± 0.7a
	BB Fafu-13	2.4 ± 1.7b	0.0 ± 0.0b	2.4 ± 1.5b	0.0 ± 0.0b	3.4 ± 0.2b	1.6 ± 0.5b
	BB Fafu-16	5.4 ± 1.3b	2.0 ± 0.7b	3.2 ± 1.0b	0.8 ± 0.6b	7.4 ± 0.9b	2.4 ± 0.2b
17 days	Control	26.0 ± 6.4a	16.6 ± 6.3a	19.4 ± 2.5a	22.6 ± 5.4a	19.2 ± 2.3a	17.4 ± 2.6a
	BB Fafu-13	2.6 ± 1.7b	0.0 ± 0.0b	2.4 ± 1.5b	0.0 ± 0.0b	5.6 ± 0.6c	3.4 ± 0.5b
	BB Fafu-16	5.4 ± 0.9b	3.6 ± 1.1b	3.0 ± 1.0b	1.6 ± 0.5b	11.0 ± 1.6b	4.6 ± 1.0b
21 days	Control	27.6 ± 8.4a	23.8 ± 6.8a	27.0 ± 4.3a	24.2 ± 3.9a	24.4 ± 2.8a	29.8 ± 4.1a
	BB Fafu-13	1.8 ± 1.2b	1.2 ± 1.0b	0.8 ± 0.6b	1.2 ± 0.8b	6.0 ± 0.8b	4.4 ± 0.5b
	BB Fafu-16	8.8 ± 1.0b	6.0 ± 0.5b	3.6 ± 2.0b	2.4 ± 0.8b	10.2 ± 1.5b	6.6 ± 1.1b

For each Asian citrus psyllid generation, mean (±SE) nymph and adult numbers followed by different letters within the same sampling date indicate significant differences among treatments at *p* = 0.05 (LSD after repeated-measures one-way ANOVA).
